# Authentically radiolabelled Mn(II) complexes as bimodal PET/MR tracers

**DOI:** 10.1186/2197-7364-2-S1-A85

**Published:** 2015-05-18

**Authors:** Christian Vanasschen, Marie Brandt, Johannes Ermert, Bernd Neumaier, Heinz H Coenen

**Affiliations:** Institute of Neuroscience and Medicine, INM-5 - Nuclear Chemistry, Forschungszentrum Jülich, Germany; Institute for Radiochemistry and Experimental Molecular Imaging, Medical Clinics, University of Cologne, Germany

The development of small molecule bimodal PET/MR tracers is mainly hampered by the lack of dedicated preparation methods. Authentic radiolabelling of MR contrast agents ensures easy access to such probes: a ligand, chelating a paramagnetic metal ion (e.g. Mn2+) and the corresponding PET isotope (e.g. 52gMn), leads to a “cocktail mixture” where both imaging reporters exhibit the same pharmacokinetics. Paramagnetic [55Mn(CDTA)]2- shows an excellent compromise between thermodynamic stability, kinetic inertness and MR contrast enhancement. Therefore, the aim of this study was to develop new PET/MR tracers by labelling CDTA ligands with paramagnetic manganese and the β+-emitter 52gMn. N.c.a. 52gMn (t1/2: 5.6 d; Eβ+: 575.8 keV (29.6%)) was produced by proton irradiation of a natCr target followed by cation-exchange chromatography. CDTA was radiolabelled with n.c.a. 52gMn2+ in NaOAc buffer (pH 6) at RT. The complex was purified by RP-HPLC and its stability tested in PBS and blood plasma at 37°C. The redox stability was assessed by monitoring the T1 relaxation (20 MHz) in HEPES buffer (pH 7.4). A functionalized CDTA ligand was synthesized in 5 steps. [52gMn(CDTA)]2- was quantitatively formed within 30 min at RT. The complex was stable for at least 6 days in PBS and blood plasma at 37°C and no oxidation occurred within 7 months storage at RT. Labelling CDTA with an isotopic 52g/55Mn2+ mixture led to the corresponding bimodal PET/MR tracer. Furthermore, a functionalized CDTA ligand was synthesized with an overall yield of 18-25%. [52g/55Mn(CDTA)]2-, the first manganese-based bimodal PET/MR tracer prepared, exhibits excellent stability towards decomplexation and oxidation. This makes the functionalized CDTA ligand highly suitable for designing PET/MR tracers with high relaxivity or targeting properties.

